# Multiple Primary Malignant Neoplasms in an Elderly Patient

**DOI:** 10.7759/cureus.2384

**Published:** 2018-03-28

**Authors:** Jai D Parekh, Shweta Kukrety, Abhishek Thandra, Carrie Valenta

**Affiliations:** 1 Internal Medicine, Creighton University Medical Center, Omaha, USA

**Keywords:** multiple primary malignant neoplasms, triple malignancy, rectal squamous cell carcinoma, renal cell carcinoma, breast cancer

## Abstract

Only a few case reports to date have described patients with three or more cancers. However, the incidence of multiple primary malignancies is increasing due to the improved survival of cancer patients, the prolonged lifespan of the general population, and better diagnostic techniques. This report describes a 73-year-old woman with primary breast, rectal squamous cell, and renal cell carcinomas. This case is unique because, in addition to having three primary malignancies, this patient had rectal squamous cell carcinoma—one of the rarest types of rectal cancer. We discuss screening and prevention of multiple malignancies and rectal squamous cell carcinoma, as well as methods for managing these patients.

## Introduction

Multiple primary malignant neoplasms (MPMN) are defined as two or more primary malignancies, in which each tumor is not an extension, recurrence, or metastasis of the other. The occurrence of multiple primary cancers in a single patient is relatively rare, although improved survival of cancer patients and a longer lifespan of the general population have increased the incidence of MPMN [1-2]. The accurate identification and management of this challenging condition have, therefore, become increasingly important.

## Case presentation

A 73-year-old Caucasian woman presented with rectal bleeding for one month. Her previous medical history included left-sided breast cancer, which was treated with radical mastectomy and adjuvant chemotherapy 18 years earlier. She reported no family history of cancer. The patient was a lifetime non-smoker and denied using alcohol or any other recreational drug. Physical examination showed that the patient was obese, with a body mass index (BMI) of 40 kg/m2. Laboratory findings were unremarkable.

**Figure 1:**
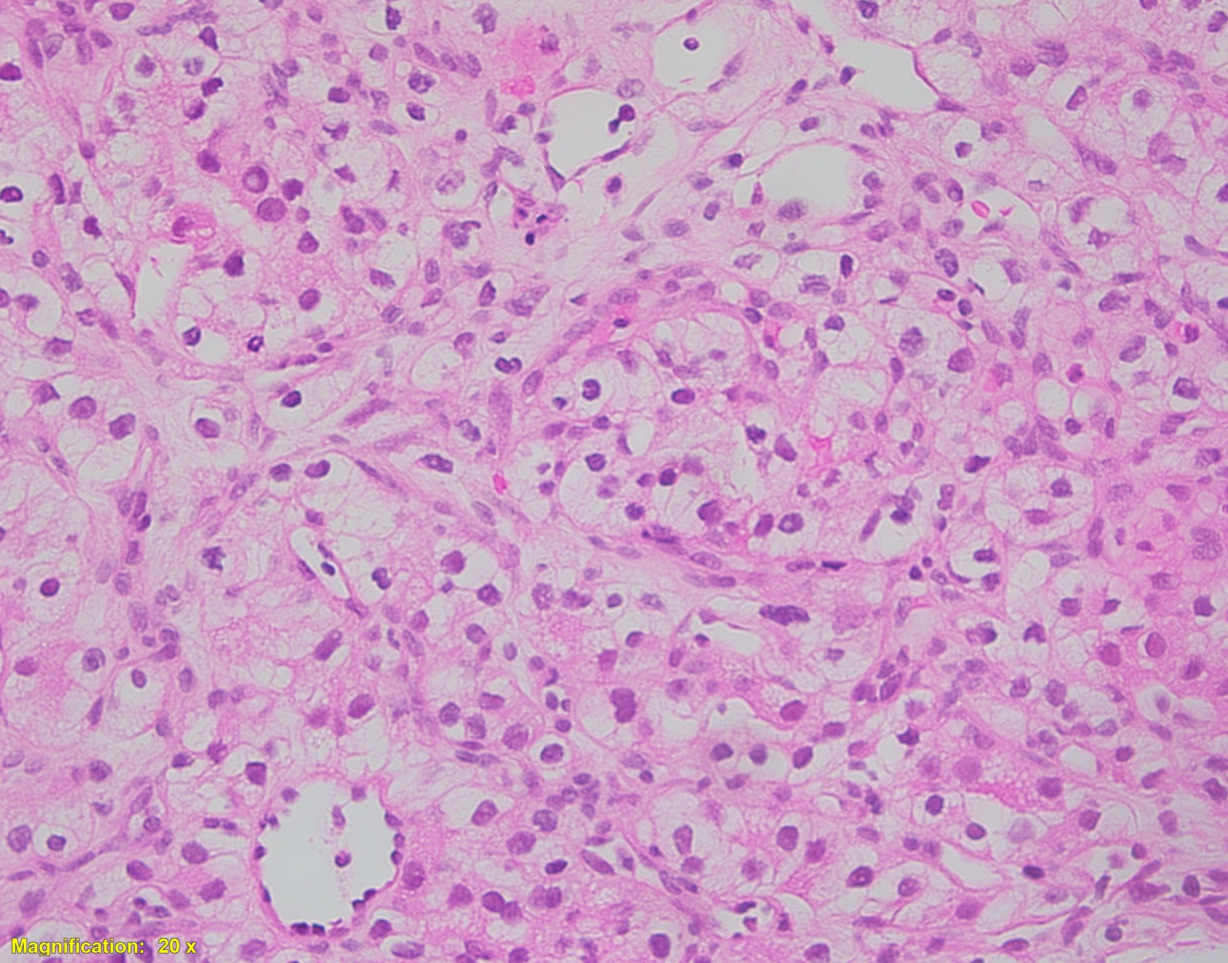
Histological examination of the resected kidney tumor Tumor cells are arranged in a solid architectural pattern with indistinct cell borders and clear cytoplasm, consistent with renal clear cell carcinoma (Hematoxylin and eosin, 200X).

The rectal bleeding was further investigated by a colonoscopy with biopsy, which revealed a rectal mass and nine colonic polyps. Histopathological evaluation showed that the rectal mass and one sigmoid polyp contained areas of invasive squamous cell carcinoma (SCC). Computed tomography (CT) of the chest, abdomen, and pelvis was performed to rule out metastatic disease. No intra-thoracic metastases were detected, but the CT image of the abdomen revealed a 9-cm solid mass on the upper pole of the right kidney, with renal biopsy showing renal cell carcinoma (RCC) (Figure 1).

The patient's rectal SCC was managed with chemoradiation, and the renal tumor was managed with right laparoscopic radical nephrectomy. Histopathologic examination of the latter tumor confirmed that it was a clear cell carcinoma, Fuhrman Grade 1-2 (Stage T2a, N0, M0). Her postoperative course was uneventful, and the patient was discharged home with close follow-up evaluations.

**Figure 2:**
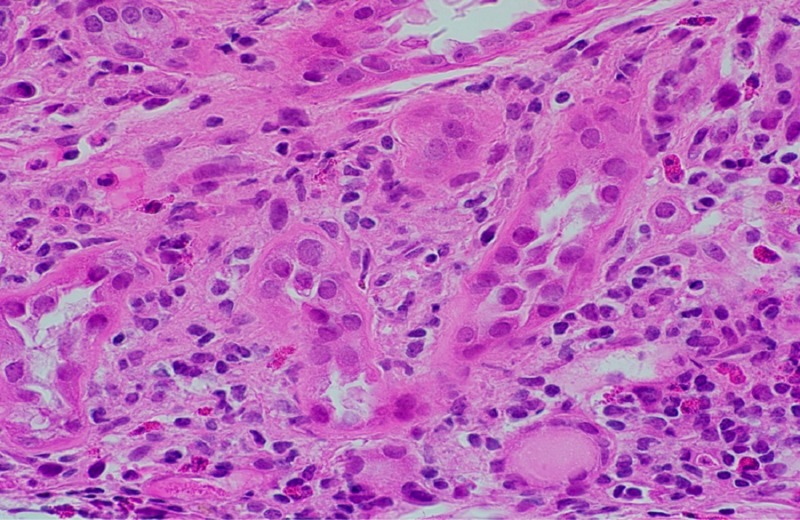
Histological examination of the kidney following surgery Diffuse interstitial edema and patchy mononuclear inflammatory cell infiltrates were consistent with acute tubulointerstitial nephritis (Hematoxylin and eosin, 600X).

Two months later, the patient returned to the hospital with concerns of weakness and fatigue. Laboratory findings showed a serum creatinine concentration of 9.4 mg/dL and a serum potassium concentration of 7.1 mmol/L. The patient underwent emergency dialysis. A CT-guided renal biopsy showed acute tubulointerstitial nephritis, hypothesized to be secondary to excessive non-steroidal anti-inflammatory drug use (Figure 2). The CT scan also demonstrated retroperitoneal lymphadenopathy encasing the distal abdominal aorta and proximal left iliac artery. The patient was started on pulse dose steroids, but her renal function failed to improve. Subsequently, the patient declined to undergo biopsy of the enlarged nodes and declined to continue receiving dialysis. She was discharged home with hospice care and passed away.

## Discussion

MPMN diagnostic criteria were established by Warren and Gates in 1932 [3]. To be considered an MPMN, each cancer must be (1) histologically different, (2) each must be definitively malignant histopathologically, and (3) the possibility of metastasis must be excluded [3].

MPMN can be further categorized into two types, synchronous and metachronous. In synchronous MPMN, all the malignant tumors develop at the same time or within six months of the first tumor. In metachronous MPMN, the second or other additional malignancy is diagnosed at least six months after the first primary tumor. Metachronous MPMN are more frequent than synchronous MPMN, with a ratio of 2.7:1 [4]. Our patient met all the diagnostic criteria for MPMN. She had synchronous rectal SCC and RCC, as well as having had breast cancer 18 years earlier. She was therefore diagnosed with metachronous triple primary neoplasms.

The prevalence of MPMN has been reported to vary from 0.73% to 16% [5]. The incidence of multiple primary cancers is increasing, with the Surveillance, Epidemiology, and End Results Program of the National Cancer Institute [6] reporting that one of six (16%) patients with a primary cancer having a second malignant neoplasm.

Multiple factors have been implicated in the pathogenesis of MPMN, including older age [2]. As the lifespan of individuals in the general population continues to increase, the incidence of multiple primary cancers will likely increase [2]. Better quality anti-neoplastic therapy has also significantly improved the survival of cancer patients, with cancer survivors having a 20% higher risk of developing a new primary cancer than the general population [1]. Anti-neoplastic therapy (radiotherapy and chemotherapy) itself is associated with an increased risk of developing a second primary malignancy. For example, patients treated with alkylating chemotherapy are at greater risk of developing acute leukemia. In addition, some patients may have an inherited predisposition to cancer. Long-term exposure of different organ systems to the action of carcinogenic factors increases susceptibility to MPMN. For example, heavy tobacco use increases the risks of developing lung, stomach, liver, kidney, uterine, cervix, and urinary bladder cancers, and excessive alcohol use is associated with malignancies of the oral cavity, pharynx, larynx, esophagus, colon, rectum, liver, and breast. Obesity is related to an increased risk of postmenopausal breast cancer, as well as endometrial, colorectal, esophageal, gallbladder, kidney, pancreatic, and thyroid cancers [7]. Our patient was obese, with a BMI of 40 kg/m2, which may have increased her risks of breast, rectal, and renal malignancies. Exposure of tissues with similar embryological origins to carcinogens may result in the development of multiple primary tumors [8]. Patients with hereditary cancer syndromes, such as multiple endocrine neoplasias, can also develop several primary cancers. Our patient, however, did not have any family history of neoplasia, making a hereditary condition unlikely.

No standard guidelines are currently available for the treatment of MPMN. Each patient should be considered individually, preferably by a multidisciplinary team. The type of each malignancy, the stage of the disease, and the patient’s overall health should be taken into account. Aggressive curative therapy should be considered for ideal treatment candidates, whereas palliative care should be offered to those who are not. Treatment should consist of the standard of care for each malignancy, including radical nephrectomy for RCC (as in our case).

There is very limited guidance on screening cancer survivors for the development of a second malignancy. Screening recommendations for the most commonly encountered second malignancies have been proposed based on expert opinions and available guidelines. For patients with a history of radiation to the chest between age 10 - 30 years, breast cancer screening is recommended with breast magnetic resonance imaging starting at age 25 years (or for women with a history of chest radiation, five to 10 years after radiation) [7]. Cancer survivors who received radiation in which the colon/rectum was included in treatment fields, screening should begin at age 35 years or 10 years after radiation [7]. Moreover, few studies have discussed the prevention of second cancers. The American Cancer Society recommends smoking cessation in patients with a prior history of radiation to the chest [7]. A systematic review found that patients with limited stage small cell lung cancer who continued to smoke were at increased risk of additional cancers [9].

Rectal SCC is a rare gastrointestinal malignancy, accounting for 0.3% of rectal cancers [10]. Current literature consists mainly of case reports and case series with no definitive guidelines on the management of these tumors. Although rectal adenocarcinomas have been treated surgically, rectal SCCs are increasingly treated with chemoradiation, similar to the definitive treatment of anal SCCs. Patients who do not respond to chemoradiation can be managed by salvage surgical treatment. Patients are generally evaluated every three months for the first two years, and every six months for an additional three years of follow-up.

## Conclusions

In conclusion, the possibility of MPMN, as well as metastasis or recurrence, should be considered in patients diagnosed with malignancies. Additional studies are required to investigate better screening practices for cancer survivors, as early detection is associated with improved outcomes. There is also an urgent need to establish evidence-based approaches to manage patients with MPMN.
